# Response to Letter to the Editor from Finsterer “Diverse Phenotypes of Mitochondrial Disease With Varying Levels of Heteroplasmy”

**DOI:** 10.1210/jcemcr/luaf128

**Published:** 2025-07-01

**Authors:** Rebecca John, Aaron Chapla, Geeta Chacko, Sangeetha Yoganathan, Maya Mary Thomas, Nihal Thomas

**Affiliations:** Department of Endocrinology, Diabetes and Metabolism, Christian Medical College, Vellore, Tamil Nadu 632004, India; Department of Endocrinology, Diabetes and Metabolism, Christian Medical College, Vellore, Tamil Nadu 632004, India; Department of Pathology, Christian Medical College, Vellore, Tamil Nadu 632004, India; Department of Pediatric Neurology, Christian Medical College, Vellore, Tamil Nadu 632004, India; Department of Pediatric Neurology, Christian Medical College, Vellore, Tamil Nadu 632004, India; Department of Endocrinology, Diabetes and Metabolism, Christian Medical College, Vellore, Tamil Nadu 632004, India; Head, Centre for Stem Cell Research, CMC Vellore (A Unit of Stem Bengaluru), Vellore, Tamil Nadu 632002, India

Dear Editor,

We are grateful to Prof Finsterer for his keen interest in our case report and his valuable insights on the subject [1]. In this letter, we would like to address some of the concerns brought forward.

We agree with the author of the letter for his valuable comments regarding the role of mitochondrial DNA (mtDNA) copy number variation, haplotype background, and variants in the nuclear gene influencing the phenotypic variability in mitochondrial disorders. The clinical manifestations in our proband's daughter were myopathy, headache, visual disturbances, cognitive decline, encephalopathy, and seizures. There was a strong family history to suggest a mitochondrial inheritance based on the family pedigree. Her laboratory evaluation revealed lactic acidosis and elevated levels of serum creatine kinase and alanine as well as a muscle biopsy revealing ragged red fibers and absence of cytochrome oxidase (COX) activity [2]. Targeted mitochondrial genome sequencing was considered as she fulfilled the diagnostic criteria for definitive mitochondrial encephalopathy with lactic acidosis and stroke-like episodes (MELAS), and targeted testing revealed a pathogenic variant A3243G in the *MT-TL1* gene [3]. The phenotypic spectrum, laboratory findings, and genotype were consistent with MELAS. The phenotypic heterogeneity could be explained by the differences in heteroplasmy, tissue distribution, and threshold effect in our proband and her daughter. The m.3243A > G variant in MELAS has been well documented to have diverse clinical phenotypes and severity [4]. However, segregation analysis in the extended family members and comprehensive genetic testing (mtDNA copy number variation, haplotype, and variants in nuclear genes) would provide additional information but were not performed in our proband's family due to the fiscal constraints. As the reviewer had rightly pointed out, comprehensive genetic analysis—including both mitochondrial and nuclear genomes—may provide important insights into the heterogeneity observed in these patients.

There was a strong family history of cardiac events as depicted in the pedigree chart (Fig. 1) in our case report [2]. We follow recommended surveillance for monitoring multisystem complications in individuals with MELAS [4]. Hence, our proband had initial visits and follow-up evaluations with electrocardiogram (ECG), and echocardiography to monitor the progression of the underlying disease and cardiac involvement. Her baseline ECG, Holter monitoring, and echocardiography did not reveal any abnormalities (normal left ventricle [LV] function, no regional wall motion abnormalities, normal LV wall thickness, normal right ventricle [RV] function, all cardiac chambers normal in size). However, a recent echocardiography revealed features that were suggestive of RV free wall thickening (measuring 8 mm), without evidence of pulmonary hypertension or significant anomalies in blood flow. There was no cardiac phenotype related to the patterns that were described in MELAS seen. A cardiac magnetic resonance imaging (MRI) scan had been planned; however, the patient had declined prioritizing the procedure as a priority, considering that she was asymptomatic.

Our proband did not have neurological manifestations up to the present time, while her daughter had central nervous system (CNS) and peripheral nervous system (PNS) involvement. As per category B in the diagnostic criteria of MELAS, high plasma or cerebrospinal fluid (CSF) lactate would be sufficient to establish a diagnosis [3]. Our proband's daughter had high blood lactate and the characteristic muscle biopsy findings; CSF lactate analysis was not carried out, considering the reluctance of the patient's family to proceed with further invasive testing. Her brain magnetic resonance spectroscopy (MRS) did not reveal a lactate peak. Identifying a lactate peak on MRS has been demonstrated to correlate with mitochondrial disease; however, this finding has not been consistently observed in every patient with mitochondrial disease [5]. However, we acknowledge that the CSF lactate is a reliable measure for the identification of CNS involvement in mitochondrial disease [6].

With regard to whether the index patient had calcification of the basal ganglia, the MRI revealed cerebellar volume loss and no evidence of basal ganglia calcification or cortical involvement. MRS did not reveal a lactate peak.

We agree that performing an autopsy in the index patient's daughter could have provided additional insights; however, culturally and logistically we could not obtain consent for autopsy at the time of death.

With regard to the expression “recurrent episodes of myopathy,” our proband's daughter had episodic muscle pain and proximal muscle weakness precipitated by a febrile illness. A diurnal variation in muscle weakness was not reported. Subsequently, she progressively developed persistent proximal muscle weakness with an elevated serum creatine kinase. Our proband has pancreatic diabetes without any evidence of CNS or PNS involvement to date while her daughter had presented with PNS and CNS involvement. The phenotypic variability in the proband and the daughter may be attributed to the diverse clinical manifestations in individuals with the m.3243A > G variant [7].

As the phenotypic spectrum and severity are highly variable in individuals with the m.3243A > G variant, our proband was counseled regarding the underlying diagnosis, the need for annual surveillance to monitor for disease progression, risk of recurrence, availability of prenatal diagnostic methods, and preimplantation genetic testing. In this instance, additional family members were either unavailable, had not yet provided informed consent, or were deceased, thereby limiting further segregation analysis. As emphasized in our case report, prospective genotypic screening of all available family members is crucial for comprehensive genetic evaluation and counseling [1].

## Data Availability

Data sharing is not applicable to this article as no datasets were generated or analyzed during the current study.

## Abbreviations

CNS: central nervous system
CSF: cerebrospinal fluid
ECG: electrocardiogram
LV: left ventricle
MELAS: mitochondrial encephalopathy with lactic acidosis and stroke-like episodes
MRI: magnetic resonance imaging
MRS: magnetic resonance spectroscopy
mtDNA: mitochondrial DNA
PNS: peripheral nervous system
RV: right ventricle
